# CRISPR/Cas12a-based approaches for efficient and accurate detection of *Phytophthora ramorum*

**DOI:** 10.3389/fcimb.2023.1218105

**Published:** 2023-06-27

**Authors:** Yufang Guo, Hongming Xia, Tingting Dai, Tingli Liu, Simon Francis Shamoun, Wu CuiPing

**Affiliations:** ^1^ Co-Innovation Center for the Sustainable Forestry in Southern China, Nanjing Forestry University, Nanjing, Jiangsu, China; ^2^ Jiangsu Provincial Key Construction Laboratory of Special Biomass Resource Utilization, Nanjing Xiaozhuang University, Nanjing, China; ^3^ Natural Resources Canada, Canadian Forest Service, Pacific Forestry Centre, Victoria, BC, Canada; ^4^ Animal, Plant and Food Inspection Center, Nanjing Customs, Nanjing, Jiangsu, China

**Keywords:** *Phytophthora ramorum*, CRISPR/Cas12a, disease diagnosis, plant destroyers, rapid detection

## Abstract

**Introduction:**

*Phytophthora ramorum* is a quarantine pathogen that causes leaf blight and shoot dieback of the crown, bark cankers and death on a number of both ornamental and forest trees, especially in North America and northern Europe, where it has produced severe outbreaks. Symptoms caused by *P. ramorum* can be confused with those by other *Phytophthora* and fungal species. Early and accurate detection of the causal pathogen *P. ramorum* is crucial for effective prevention and control of Sudden Oak Death.

**Methods:**

In this study, we developed a *P. ramorum* detection technique based on a combination of recombinase polymerase amplification (RPA) with CRISPR/Cas12a technology (termed RPACRISPR/ Cas12a).

**Results:**

This novel method can be utilized for the molecular identification of *P. ramorum* under UV light and readout coming from fluorophores, and can specifically detect *P. ramorum* at DNA concentrations as low as 100 pg within 25 min at 37°C.

**Discussion:**

We have developed a simple, rapid, sensitive, unaided-eye visualization, RPA CRISPR/Cas12a-based detection system for the molecular identification of *P. ramorum* that does not require technical expertise or expensive ancillary equipment. And this system is sensitive for both standard laboratory samples and samples from the field.

## Introduction

*Phytophthora* is a genus of plant pathogens that can cause significant harm to plants, for example, leaf necrosis, shoot blight, and fruit rot and even cause plant death (Erwin and Ribeiro, 2002; Jung et al., 2011; Jung et al., 2016; Kroon et al., 2012). Recently, European scientists have found *P. ramorum* from the laurosilva forests between Indochina and southwest Japan (Jung et al., 2021). *Phytophthora ramorum* is a highly damaging pathogen that affects 70 species of trees and shrubs around the world, which was first observed attacking *Rhododendron* and *Viburnum* species in Germany and the Netherlands, and causing widespread devastation through diseases such as sudden oak death, and ramorum blight. (Werres et al., 2001; Grünwald et al., 2008; Brasier and Webber, 2010; Kamoun et al., 2015). This pathogen has now become established in forests on the central coast of California and is causing widespread destruction of native oak trees (*Notholithocarpus densiflora*, *Quercus agrifolia*, *Q. kelloggii*, and *Q. parvula* var. *shrevei*) (Kroon et al., 2004). Nowadays, *P. ramorum* has been listed as the key quarantine regulated pathogen in North American, European and Asian countries and regions around the world (Blomquist et al., 2020). Although there have been no documented cases of *P. ramorum* causing sudden oak death in China, it is still regarded as a significant threat to forestry and has been included on China’s quarantine pathogen list since 2009.

Controlling diseases caused by *P. ramorum* is a challenging task. Therefore, it is crucial to achive early detection of *P. ramorum* in order to minimize its impact. The current methods to identify *P. ramorum* by morphology are time-consuming, insensitive, require high conditions and often produce inaccurate results, limiting their effectiveness (Cooke et al., 2007; O’Brien et al., 2009; Yang et al., 2017; Yang et al., 2023; Wang et al., 2023). Diverse molecular techniques have been developed for detection and identification of plant pathogenic Oomycetes (Cooke et al., 2000). Currently, there are several molecular detection techniques available for identifying *P. ramorum*, including PCR, real-time PCR, and nested PCR (Kroon et al., 2004; Tomlinson et al., 2005; Gagnon et al., 2014). However, the use of specialized equipment for sample treatment, amplification reaction, and result output can limit the applicability of these methods for on-site disease diagnosis. To address this issue, researchers have developed and implemented several molecular detection technologies based on isothermal amplification reactions, such as sequence-based amplification (NASBA), helicase-dependent amplification (HAD), rolling circle amplification (RCA), loop-mediated isothermal amplification (LAMP) and recombinase polymerase amplification (RPA) (Tomlinson et al., 2010; Hieno et al., 2021). Unlike DNA extraction-based strategies, RPA is an isothermal nucleic acid amplification technique that overcomes many of the associated limitations (Piepenburg et al., 2006). The compatibility of RPA is excellent since it can be widely combined with other analytic techniques, including gel electrophoresis, enzyme-linked immunosorbent assay (Toldrà et al., 2018), real-time fluorescent quantitative (Srivastava et al., 2019), and lateral-flow dipstick (LFD) (Rosser et al., 2015).

The CRISPR-Cas system, which involves Cas enzymes and guide RNA, has recently gained significant attention as a diagnostic tool due to its ability to specifically target genes (Kadam et al., 2023). The first biological evidence that CRISPR-Cas systems play a role in adaptive immunity was reported in 2007 when *S. thermophilus* CRISPR loci were shown to acquire novel spacers derived from the invasive phage DNA (Barrangou et al., 2007). This technique allows for precise genetic engineering by cleaving the target DNA or RNA based on the sequence of the guide RNA (gRNA). Additionally, some Cas proteins, such as Cas12a and Cas13a, have been found to exhibit trans-cleavage activity, which involves cleaving surrounding single-stranded DNA or RNA upon the binding of the Cas-gRNA complex to the target (Barrangou et al., 2007; Wiedenheft et al., 2012; Chen et al., 2018; Li et al., 2018; Kim et al., 2021; Kadam et al., 2023), including pathogen detection (Wang et al., 2020). In the CRISPR/Cas12a system, a CRISPR RNA (crRNA)/Cas12a complex specifically recognizes and guides the cleavage of target double-stranded DNA (dsDNA) *via* a protospacer adjacent motif (PAM) (Chen et al., 2018; Xiao et al., 2021). After the *in vitro* reaction, the cleavage products can be observed using a fluorescence reader. This requires the introduction of a single-stranded DNA reporter that has been labeled with both a fluorophore and quencher (Xiong et al., 2020; Zhu et al., 2021; Li et al., 2022). The effectiveness and sensitivity of the CRISPR/Cas12a assay are very low when the assay is used alone (Chen et al., 2018; Li et al., 2018; Xiao et al., 2021). To address this, an assay combining RPA with CRISPR/Cas12a (RPA-CRISPR/Cas12a)-based detection has recently been developed and applied for the detection of viral pathogens (for example *SARS-CoV-2*, Bai et al., 2019), bacterial pathogens (for example *Escherichia coli*, Chen et al., 2020), and fungal pathogens (for example *Elsinoë fawcettii*, Shin et al., 2021b).

In this research, we have established a system that can effectively detect *P. ramorum*. The method involves a quick 10-minute DNA extraction process, followed by RPA-mediated amplification of the *Pr52094* gene within 20 minutes at 37°C. This is then followed by CRISPR/Cas12a-based detection, which takes only 5 minutes at 37°C. The results can be visualized through green fluorescence under a Blue LED Transilluminator with a wavelength of 470 nm or detected using a multifunctional microplate reader with an excitation wavelength of 485 nm and an emission wavelength of 520 nm. We have confirmed the method’s feasibility, as well as its analytical sensitivity and specificity, by testing artificially inoculated samples. This is a significant development in the rapid detection of *P. ramorum* and its associated diseases.

## Materials and methods

### Maintenance of isolates and DNA extraction

The types of *P. ramorum* strains and the isolates of fungal, oomycete, and *Bursaphelenchus* species that were examined in this study are listed in **Table 1**. The *P. ramorum* isolates were obtained from the Animal, Plant, and Food Inspection Center of Nanjing and Shanghai Customs. The other *Phytophthora* species and fungal isolates used in this study are kept in a collection at the Department of Plant Pathology, Nanjing Forestry University (NFU) in China. All isolates were identified using both morphological and molecular biology methods. Fungal isolates were grown on potato dextrose agar (PDA) at a temperature of 25°C in the dark for 3 to 5 days. Isolates of oomycete species were cultured on 10% clarified V8 juice agar at a temperature of 18 to 25°C in the dark (Xiao et al., 2023). *Bursaphelchus xylophilus* and *B. mucronatus* were propagated for one generation using the mycelia of *Botrytis cinerea* at 25°C for 4 to 5 days. Genomic DNA (gDNA) was extracted from all the isolates using the DNA secure Plant Kit (Tiangen Biotech, Beijing, China). The extracted gDNA was quantified using a NanoDrop 1000c spectrophotometer (Thermo Fisher Scientific, Massachusetts, USA) and diluted accordingly. All DNA samples were stored at a temperature of -20°C until use.

**Table 1:** Information and Crisp-cas12a deteciton results of Phytophthora and other oomycete and fungal isolates used in this study.

Number	*Species*	Isolate	Origin		Crisp-cas12a deteciton results
			Host/substrate	Source	
1	*P. ramorum*	EU1 2275	*Quercus palustris*	United Kingdom	+
2	*P. vignae*	CPHST BL 30	*Vigna* sp.	M. D. Coffey	**−**
3	*P. melonis*	PMNJHG1	*Cucumis sativus*	JS, China	**−**
4	*P. melonis*	PMNJHG2	*Cucumis sativus*	JS, China	**−**
5	*P. melonis*	PMNJHG3	*Cucumis sativus*	JS, China	**−**
6	*P. melonis*	PMNJDG1	*Benincasa hispida*	JS, China	**−**
7	*P. melonis*	PMNJDG2	*Benincasa hispida*	JS, China	**−**
8	*P. melonis*	PMNJDG3	*Benincasa hispida*	JS, China	**−**
9	*P. fragariae*	CBS209.46	*Fragaria × ananassa*	England, UK	**−**
10	*P. rubi*	CBS 967.95	*Rubus idaeus*	Scotland, UK	**−**
11	*P. cambivora *	CBS 248.60	*Castanea sativa*	USA	**−**
12	*P. cambivora *	Pc1	*Malus domestica* Borkh	SH, China	**−**
13	*P. parvispora*	CBS132771	*Arbutus unedo*	Italy	**−**
14	*P. parvispora*	CBS132772	*Arbutus unedo*	Italy	**−**
15	*P.cinnamomi*	Pci1	*Pinus* sp.	AH,China	**−**
16	*P.cinnamomi*	Pci2	*Rhododendron simsii*	JS, China	**−**
17	*P.cinnamomi*	Pci3	*Cedrus deodara*	JS, China	**−**
18	*P.cinnamomi*	Pci4	*Camellia oleifera* Abel.	JS, China	**−**
19	*P.cinnamomi*	Pci5	*Pinus* sp.	JS, China	**−**
20	*P.cinnamomi*	Pci6	*Rhododendron simsii*	AH,China	**−**
21	*P.cinnamomi*	Pci7	*Rhododendron simsii*	SD, China	**−**
22	*P.cinnamomi*	Pci8	*Cedrus deodara*	SD, China	**−**
23	*P.cinnamomi*	Pci9	*Cedrus deodara*	AH,China	**−**
24	*P.cinnamomi*	Pci10	*Pinus* sp.	SD, China	**−**
25	*P. citricola*	Pcit	*Rhododendron pulchrum*	JS, China	**−**
26	*P. cactorum*	C1	*Malus pumila*	JS, China	**−**
27	*P. cactorum*	C2	*Malus pumila*	JS, China	**−**
28	*P. cactorum*	C3	*Rosa chinensis*	JS, China	**−**
29	*P. infestans*	Pi1	*Solanum tuberosum*	FJ, China	**−**
30	*P. infestans*	Pi2	*Solanum tuberosum*	YN, China	**−**
31	*P. nicotianae*	Pn1	*Nicotiana tabacum*	FJ, China	**−**
32	*P. nicotianae*	Pn2	*Lycopersicum* sp.	JS, China	**−**
33	*P. nicotianae*	Pn3	*Sophora sinensis*	JS, China	**−**
34	*P. nicotianae*	Pn4	*Citrus* sp.	JS, China	**−**
35	*P.tentaculata*	*Pt1*	*Aucklandia lappa*	YN, China	**−**
36	*P. pini*	*Ppini1*	*Rhododendron pulchrum*	*JS, China*	**−**
37	*P. pini*	*Ppini2*	*R. pulchrum*	*JS, China*	**−**
38	*P. capsici*	*Pc1*	*Capsicum annuum*	*JS, China*	**−**
39	*P. capsici*	*Pc2*	*Capsicum annuum*	*YN, China*	**−**
40	*P. capsici*	*Pc3*	*Capsicum annuum*	*SH, China*	**−**
41	*P.colocasiae*	*Pcol1*	*Colocasia esculenta (L.) Schott*	*YN,China*	**−**
42	*P.citricola*	*Pcitr1*	*Persea americana*	*JS, China*	**−**
43	*P. plurivora*	*Pplu1*	*Manihot esculenta*	*HN,China*	**−**
44	*P. ilicis*	CBS114348	*Ilex aquifolium*	Netherlands	**−**
45	*P. palmivora*	*Pp1*	*Iridaceae*	YN, China	**−**
46	*P. quercetorum*	15C7	*Soil*	USA	**−**
47	*P. castaneae*	CBS587.85	*Soil*	Taiwan	**−**
48	*P. megasperma*	CBS305.36	*Matthiola incana*	USA	**−**
49	*P. mississippiae*	57J3	*Irrigation water*	Mississippi, USA	**−**
50	*P. drechsleri*	CBS 292.35^T^	*Beta vulgaris* var*. altissima*	California (CA), USA	**−**
51	*P. drechsleri*	ATCC 56353	*Citrus sinensis*	Australia	**−**
52	*P. hibernalis*	CBS 270.31	*Cirrus sinensis*	USA	**−**
53	*P. syringae*	ATCC 34002	*Citrus* sp.	CA	**−**
54	*P. lateralis*	CBS168.42	*Cedrus deodara*	Canada	**−**
55	*P. medicaginis*	ATCC 44390	*Medicago sativa*	USA	**−**
56	*P. boehmeriae*	Pb1	*Boehmeria nivea*	JS, China	**−**
57	*P. boehmeriae*	Pb2	*Gossypium* sp.	JS, China	**−**
58	*P. boehmeriae*	Pb3	*B. nivea*	JS, China	**−**
59	*P. boehmeriae*	Pb4	*Gossypium* sp.	JS, China	**−**
60	*P. quercina*	CBS 789.95	*Quercus petraea*	Australia	**−**
61	*Phytophthora sojae (R2)*	R2	*Glycine max*	B. M. Tyler	**−**
62	*Phytopythium litorale*	PC-dj1	*Rhododendron simsii*	JS, China	**−**
63	*P. helicoides*	PH-C	*Rhododendron simsii*	JS, China	**−**
64	*P. helicoides*	PF-he2	*Photinia × fraseri* Dress	JS, China	**−**
65	*P. helicoides*	PF-he3	*Photinia × fraseri* Dress	JS, China	**−**
66	*Pythium ultimum*	Pul1	*Citrus sinensis*	JS, China	**−**
67	*Py. spinosum*	Psp1	*Oryza sativa* L.	JS, China	**−**
68	*Py. aphanidermatum*	Pap1	*Nicotiana tabacum*	JS, China	**−**
69	*Fusarium oxysporium*	Fox1	*Gossypium* sp.	JS, China	**−**
70	*F. solani*	Fso1	*Gossypium* sp.	JS, China	**−**
71	*F. solani*	Fso2	*Glycine max*	JS, China	**−**
72	*F. circinatum*	A045-1	*Pinus sp.*	SH, China	**−**
73	*F. fujikuroi*	Ffu1	*Oryza sativa*	JS, China	**−**
74	*F. graminearum*	Fgr1	*Triticum aestivum*	JS, China	**−**
75	*F. acuminatum*	Fac1	*Rhizophora apiculata*	SC, China	**−**
76	*F. asiaticum*	Fas1	*Triticum aestivum*	JS, China	**−**
77	*F. avenaceum*	Fav1	*Glycine max*	JS, China	**−**
78	*F. culmorum*	Fcu1	*Glycine max*	SC, China	**−**
79	*F. commune*	Fco1	Soil	HLJ, China	**−**
80	*F. equiseti*	Feq1	*Glycine max*	JS, China	**−**
81	*F. lateritium*	Flat1	Soil	JS, China	**−**
82	*F. moniforme*	Fmo1	*Oryza sativa*	JS, China	**−**
83	*F. nivale*	Fniv	*Triticum aestivum*	JS, China	**−**
84	*F. proliferatum*	Fpr1	*Pinus sp.*	JS, China	**−**
85	*F. incarnatum*	IL3HQ	*Medicago sativa*	JS, China	**−**
86	*Colletotrichum truncatum*	Ctr1	*Glycine max*	JS, China	**−**
87	*C. glycines*	Cgl1	*Glycine max*	JS, China	**−**
88	*C. orbiculare*	Cor1	*Citrullus lanatus*	JS, China	**−**
89	*Verticilium dahlia*e	Vda1	*Gossypium* sp.	JS, China	**−**
90	*Rhizoctonia solani*	Rso1	*Gossypium* sp.	JS, China	**−**
91	*Magnaporthe grisea*	Guy11	*Oryza sativa*	Japan	**−**
92	*Endothia parasitica*	Epa1	*Castanea mollissima*	JS, China	**−**
93	*Bremia lactucae*	Bla1	*Lactuca sativa*	JS, China	**−**
94	*Aspergillus flavus*	NJC03	*Actinidia chinensis*	SX, China	**−**
95	*Botrytis cinerea*	Bci1	*Cucumis sativus*	JS, China	**−**
96	*Alternaria alternata*	Aal1	Soil	JS, China	**−**
97	*Tilletia indica*	Tin1	*Triticum aestivum*	JS, China	**−**
98	*Diaporthe mahothocarpus*	DT1	*Kerria japonica*	JS, China	**−**
99	*D. sapindicola*	WHZ3	*Sapindus mukorossi*	JS, China	**−**
100	*Botryosphaeria dothidea*	Bci1	*Koelreuteria paniculata*	JS, China	**−**
101	*Bursaphelenchus xylophilus*	Js-1	*Pinus thunbergii*	JS, China	**−**
102	*B.mucronatus*	Bmucro	*Pinus* sp.	JS, China	**−**

a Isolate identification abbreviations: CBS, Centraalbureau voor Schimmelcultures Fungal Biodiversity Centre, Utrecht, The Netherlands; ATCC, American Type Culture Collection, Manassas, Virginia, USA.

b Abbreviations of provinces in China: JS = Jiangsu province; YN = Yunnan province; FJ = Fujian province; SD = Shandong province; GZ = Guizhou province; NMG = Neimenggu province; HLJ = Heilongjiang province; JX = Jiangxi province; SX = Shanxi province; HN = Hunan province; SH = Shanghai.

c Positive (+) or negative (−) reaction result in the RPA-CRISPR/Cas12a assay for detecting F. circinatum.

### Design of RPA primers, crRNA, and the ssDNA reporter

The *Pr52094* gene was selected as a target sequence for designing *P. ramorum*-specific RPA primers (*Pr52094*RPA-F:CAGTCCGACAGTGAGCAACTATAGTATCTCCGAG 102-135 nt, *Pr52094*RPA-R:CGTACTCTTGGAGAGTCAAGGCCCATCTGTG 268-298 nt) based on comparative whole-genome sequence analysis among *Phytophthora* species (Xu et al., 2022). The RPA primers were designed based on the recommendation listed in the manual of the TwistAmp DNA Amplification Kit (https://www.twistdx.co.uk/docs/default-source/RPA-assay-design/twistamp-assay-design-manual-v2-5.pdf?sfvrsn=29). Primer details are listed in **Supplementary Figure S1**.

The CHOPCHOP web tool (http://chopchop.cbu.uib.no/) was used to design the crRNA probe (Zhao et al., 2022). Two factors must be considered when designing the crRNA(crRNA : UAAUUUCUACUAAGUGUAGAUCGCCCAUAGAGAUGCGGUCG)(**Figure S1**), namely, the crRNA sequence must not overlap with that of the RPA primers, and the crRNA must target a conserved region of the RPA amplicon (**Supplementary Figure S1**). The single-stranded DNA (ssDNA) reporter used in the study had a 6-FAM label at its 5’ end and was blocked with the BHQ-1 quencher at its 3’ end (5’ 6-FAM-TTATT-BHQ-1 3’) (Chen et al., 2018; Li et al., 2018). Both the crRNA and ssDNA reporter were synthesized by GenScript in Nanjing, China and stored at a temperature of -20°C until further use.

### The RPA-CRISPR/Cas12a assay

**Figure 1:**
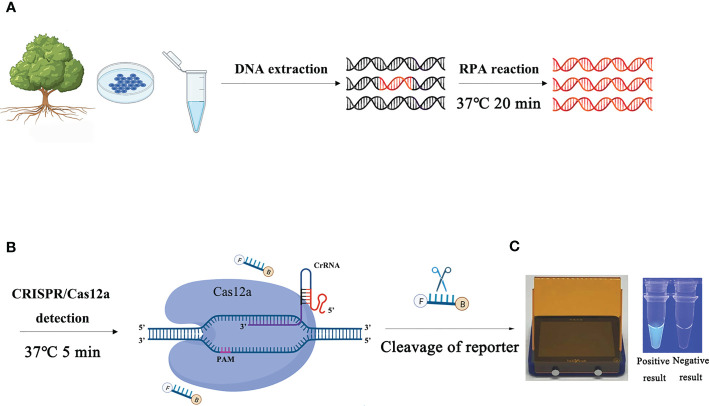
Schematic diagram of the RPA-CRISPR/Cas12a assay for the detection of *Phytophthora ramorum*. **(A)** Recombinase polymerase amplification (RPA). **(B)** Cas12a protein can combine with each amplicon and target-specific crRNA to form a complex with an indiscriminate ssDNA cleavage activity. The FAM-labeled ssDNA reporter is cleaved and produces visible green fluorescence under excitation at a wavelength of 470 nm. **(C)** Positive result: visible green fluorescence. Negative result: non-visible green fluorescence.

The process of detecting *P. ramorum* using the RPA-CRISPR/Cas12a assay is illustrated in **Figure 1**. The assay takes only 20 minutes and involves a two-step process, with 15 minutes allocated for the RPA reaction and 5 minutes for the CRISPR/Cas12a assay. The first step uses a pair of primers, Pr52094RPA-F/Pr52094RPA-R, to amplify the *Pr52094* gene of *P. ramorum* through recombinase polymerase amplification (RPA) in just 15 minutes. The second step employs the CRISPR/Cas12a system to detect and visualize the presence of the target gene within 5 minutes. RPA reactions were performed in 50-µl reactions according to the quick guide of the Test Strip Kit (Lesunbio, WuXi, China). Each reaction mixture initially contained 2 µl of each forward and reverse primer (Pr52094RPA-F/Pr52094RPA-R, 10 µM), 25 µl of rehydration buffer (supplied with the kit), gDNA (100 ng), and 16 µl of double-distilled H_2_O (total 47 µl). For each sample, a centrifugation step was performed at 4,000 rpm for 5 seconds, followed by the addition of 3 µl of activator to the cap of the reaction tube, which was then carefully tightened. The activator was added to the premix by centrifuging at 4,000 rpm for 5 seconds. Reactions were conducted at 37°C, and after 5 minutes, the samples were mixed by hand, centrifuged at 4,000 rpm for 5 seconds, and further incubated for 20 minutes at 37°C. **25 minutes were the actual time spent for the detection test.** To eliminate false positives, each set of reactions included a no-template control (NTC) and a positive control (PTC). The RPA products were analyzed using 1.5% agarose gel electrophoresis or the CRISPR/Cas12a system. The appropriate assay conditions were determined using the RPA amplification product as the template and different concentrations of crRNA (40 nM, 80 nM, 300 nM, 0.5 µM, 0.6 µM, 1 µM, 2 µM, 5 µM, and 10 µM) and ssDNA reporter (40 nM, 500 nM, 1.4 µM, 2 µM, 5 µM, and 10 µM).

The CRISPR/Cas12a assay was conducted in 50-µl reactions consisting of various components. These included 38 µl of double-distilled H2O (ddH2O), 5 µl of 10× Reaction Buffer 1, 3 µl of crRNA (at a concentration of 1 µM), 1 µl of Cas12a (at a concentration of 2 µM) (Magigen, Guangzhou, China), 1 µl of the ssDNA reporter (at a concentration of 10 µM), and 2 µl of RPA products. Once the reaction mixture was assembled, it was immediately incubated at 37°C for 5 minutes. Fluorescence was then observed using a Blue LED Transilluminator (Baisai Ltd, Shanghai, China) at a wavelength of 470 nm, or detected using a multifunctional microplate reader (λex: 485 nm, λem: 520 nm). All template concentrations and assays were evaluated three times, and statistical analysis was performed using GraphPad Prism 8 software (GraphPad Software Inc., San Diego, CA, USA). All template concentrations and all assays were evaluated three times. The CRISPR/Cas12a assay was repeated three times and we got three results, then we use STDEVP(number1,number2, number 3) to get the standard error. Statistical analysis was performed using GraphPad Prism 8 software (GraphPad Software Inc., San Diego, CA, USA). The experimental group and control group were compared by performing the Student’s t-test for a difference analysis by calculating P value. P <0.05 (*) was considered statistically significan.

### Conventional PCR assay

A conventional PCR was conducted in 50-µl reactions using the following components: 25 µl of Prime STAR Max Premix 2×(Takara Bio, DaLian, China), 21 µl of dd H2O, 100 ng of purified gDNA, and 1 µl each of forward and reverse primers (10 µM). The thermal cycling program comprised 94°C for 3 min, followed by 33 cycles of 94°C for 30 s, 60°C for 30 s, and 72°C for 45 s, and a final extension step of 72°C for 10 min. The amplification was performed using an Applied Biosystems Veriti Dx 96-Well Thermal Cycler (Thermo Fisher Scientific). Each set of reactions included a positive template control (PTC) and a no-template control (NTC). After amplification, the PCR products were subjected to electrophoresis on a 1.5% agarose gel at 130 V for approximately 30 min and then visualized under a UV transilluminator. The PCR assay was repeated three times.

### Determining the specificity and sensitivity of the RPA-CRISPR/Cas12a assay

Conducting a comparative analysis to assess the specificity and sensitivity of the RPA-CRISPR/Cas12a assay for detecting *P. ramorum*. The study involved testing the assay against conventional PCR under optimal conditions using purified isolate gDNA (100 ng) as a template for both methods. The assay’s specificity was determined by testing against the isolates listed in **Table 1**, with a positive control (*P. ramorum* isolate, 100 ng) and a NTC included in each set of reactions. We carried out this experiment three times.

To evaluate the assay’s sensitivity, we utilized nine serial dilutions of *P. ramorum* gDNA (100 ng, 10 ng, 1 ng, 100 pg, 10 pg, 1 pg and 100 fg) as templates for both the conventional PCR and RPA-CRISPR/Cas12a assays. We included a NTC in each set of reactions and performed three replicates for each template concentration in both methods.

### Detection of *Phytophthora ramorum* in artificially inoculated *Rhododendron × pulchrum* using the RPA-CRISPR/Cas12a assay

To detect *P. ramorum*, leaves of *Rhododendron × pulchrum* (NJFU, Nanjing, China) were used in an RPA-CRISPR/Cas12a assay. Before inoculation, the leaves were washed with distilled water for 10 minutes, then immersed in 70% ethanol for 10 seconds, and finally rinsed with distilled water. *P. ramorum* strains were cultured on V8 agar medium in the dark at 25°C, and a 5-day-old V8 plug (0.5 × 0.5 cm) from the actively growing area of the colonies was placed onto the wound site in three replicate leaves. Three leaves were treated with a sterile PDA plug as a control.

All leaves were then placed in a container with two layers of wet filter paper, stored in a dark incubator at 25°C with 100% relative humidity, and subjected to a 12-hour light/12-hour dark photoperiod. After 120 hours, crude DNAs were extracted from 100 mg stem segments at the inoculation site using NaOH lysate solution, which consisted of 20 mM sodium hydroxide, 5% polyethylene glycol 200, and 5% dimethyl sulfoxide. To extract the DNA, 1mL NaOH lysate and 100 mg of *P. ramorum*-inoculated samples in liquid nitrogen powder were mixed vigorously and incubated at room temperature (25°C) for 10 minutes. The tubes were tapped three times during incubation. After incubation, 2 µL of lysate solution was used as the RPA assay template, with *P. ramorum* (100 ng) and dd H_2_O serving as the PTC and NTC, respectively. This experiment was performed three times.

## Results

### Optimizing the RPA-CRISPR/Cas12a assay for the detection of *P. ramorum*

**Figure 2:**
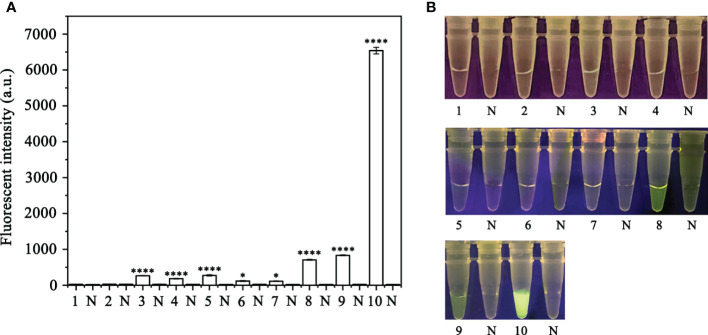
Screening for the optimal crRNA and ssDNA reporter concentrations for the RPA-CRISPR/Cas12a assay. The crRNA and ssDNA reporter concentrations were set. 1: 40 nM crRNA, 40 nM ssDNA reporter; 2: 80 nM crRNA, 40 nM ssDNA reporter; 3: 300 nM crRNA, 40 nM ssDNA reporter; 4: 300 nM crRNA, 500 nM ssDNA reporter; 5: 2 µM crRNA, 2 µM ssDNA reporter; 6: 1 µM crRNA, 10 µM ssDNA reporter; 7: 5 µM crRNA, 10 µM ssDNA reporter; 8: 5 µM crRNA, 5 µM ssDNA reporter; 9: 2 µM crRNA, 10 µM ssDNA reporter; 10: 10 µM crRNA, 10 µM ssDNA reporter; All other parameters were identical. N: negative (no-template) controls (NTC). **(A)** Fluorescence detection using a multifunctional microplate reader (λ_ex_: 485 nm, λ_em_: 520 nm), *P <0.05 was considered statistically significant. ****P <0.0001 indicates that the difference between fluorescence and non fluorescence is very significant. **(B)** Detection of visible green fluorescence under the Blue LED Transilluminator at a wavelength of 470 nm.

**Figure 3:**
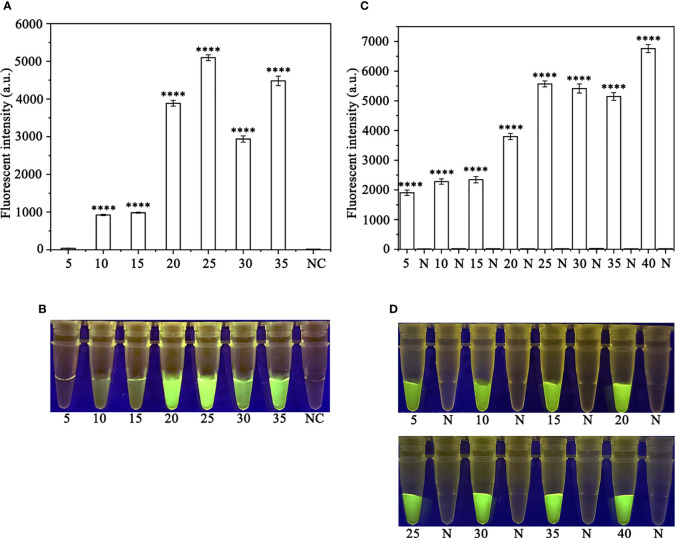
Optimizing the RPA reaction time and the Cas12a cleavage time. **(A, B)** 5-35 (RPA reaction time): 5, 10, 15, 20, 25, 30, and 35 min; N and NC: negative (no-template) control (NTC). **(C, D)** 5, 10, 15, 20, 25, 30, 35 and 40 (Cas12a cleavage time): 5, 10, 15, 20, 25, 30, 35, and 40 min; N: negative control. **(A, C)** Fluorescence detection using a multifunctional microplate reader (λ_ex_: 485 nm, λ_em_: 520 nm), P<0.0001(****) was considered statistically significant. **(B, D)** Detection of visible green fluorescence under the Blue LED Transilluminator at a wavelength of 470 nm.

In order to optimize the concentrations of the crRNA probe and the ssDNA reporter for the RPA-CRISPR/Cas12a assay, various concentrations were tested and the results indicated that the fluorescence intensity plateaued at 10 µM for both the crRNA probe and the ssDNA reporter (**Figure 2**). To improve the efficiency of the assay and minimize the time required, different RPA reaction times (5, 10, 15, 20, 25, 30, and 35 minutes) and Cas12a cleavage times (5, 10, 15, 20, 25, 30, 35, and 40 minutes) were tested using a constant amount of *P. ramorum* gDNA template (100 ng). The results revealed that fluorescence could be clearly observed after 20 minutes (**Figures 3A, B**). After evaluating the results, 5 minutes was determined to be the optimal time for both the RPA reaction and Cas12a-mediated cleavage (**Figures 3C, D**). This approach enabled the assay to be completed more rapidly without compromising its effectiveness.

### Specificity of RPA-CRISPR/Cas12a assay in rapid detection of *P. ramorum*

**Figure 4:**
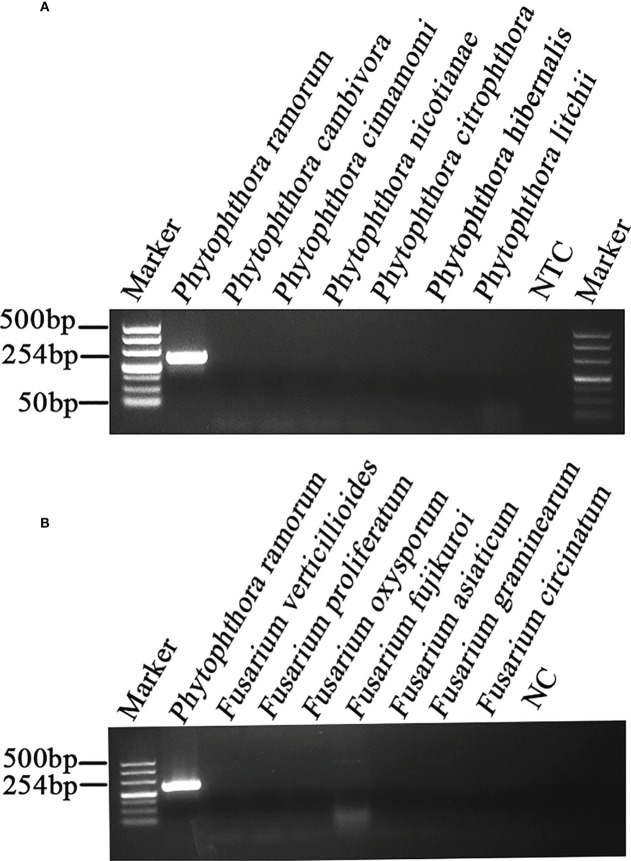
Primers designed based on the *Pr52094* gene were screened for specificity using conventional PCR assays, and detected by 1.5% agarose gel electrophoresis. PCR amplicons (254 bp) were only detected in the *P. sojae* sample, indicating specificity regarding the detection of *P. ramorum* DNA. The *Pr52094* gene was found to be specific for detecting *P. ramorum*. Marker DL500 (Takara Shuzo, Shiga, Japan). NC (Negative control, double-distilled H_2_O). **(A)** Specificity evaluation of the PCR assay based on the target gene *Pr52094* among *Phytophthora* species. Approximately 254 bp-long PCR amplicons were detected in reactions containing gDNA of *P. ramorum* isolates. No PCR amplicons were detected among reactions containing gDNA of other *Phytophthora* species or NTCs. **(B)** Specificity evaluation of the PCR assays based on *Pr52094* using other fungal. Approximately 254 bp-long PCR amplicons were detected in reactions containing gDNA of *P.ramorum* isolates. No PCR amplicons were detected among reactions containing gDNA of other fungal, or NTCs." after "NC (Negative control, double-distilled H2O).

**Figure 5:**
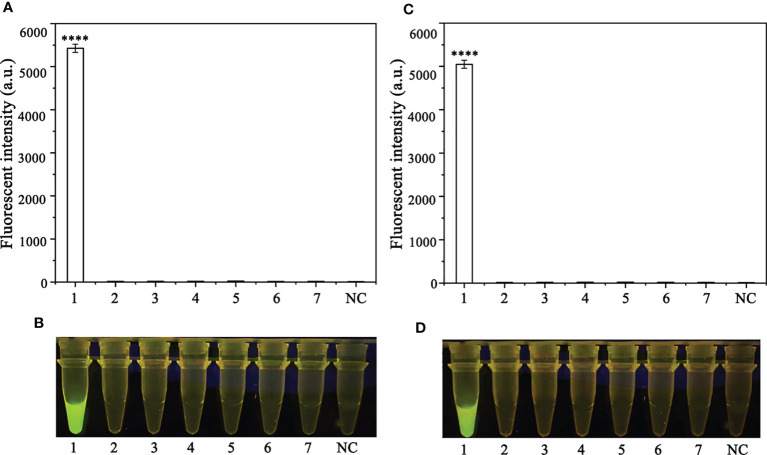
Evaluation of the specificity of the novel RPA-CRISPR/Cas12a assay. **(A, B)** Evaluation using 1: *Phytophthora ramorum*, 2: *P. pini*, 3: *P. syringae*, 4: *P. hydrogena*, 5: *P.parasitica*, 6: *P. drydrogena*, 7: *P. cryptogea* and 8: negative (no template) control (NC). **(C, D)** Evaluation using 1: *P. sojae*, 2: *Alternaria alternata*, 3: *Botrytis cinerea*, 4: *Colletotrichum truncatum*, 5: *Endothia parasitica*, 6: *Fusarium oxysporium*, 7: *Fusarium solani*, and 8: negative (no template) control (NC).

A PCR amplification product of approximately 254 bp was amplified in the RPA reaction from the gDNA of *P. ramorum* with the primers Pr52094RPA-F and Pr52094RPA-R. No PCR amplicons were detected in the reactions with gDNA of *P. cambivora*, *P. cinnamomi*, *P. nicotianae*, *P. citrophthora*, *P. hibernalis*, *P. litchii*, *Fusarium verticillioides*, *F. proliferatum*, *F. oxysporum*, *F. fujikuroi*, *F. asiaticum*, *F. graminearum*, *F. circinatum* and the NC (**Figure 4**). The researchers used the DNA of 32 different *Phytophthora* species, 5 oomycete species, 31 fungal species, and 2 *Bursaphelenchu*s species to test the specificity of their developed RPA-CRISPR/Cas12a assay (**Table 1**). The results demonstrated that only when *P. ramorum* gDNA was used as the RPA reaction template, could visible green fluorescence be detected using a Blue LED Transilluminator at a 470-nm wavelength (**Figures 5B, D**) or a multifunctional microplate reader with λex: 485 nm, λem: 520 nm (**Figures 5A, C**). These findings indicate that the RPA-CRISPR/Cas12a assay is highly specific for *P. ramorum*.

### Sensitivity of the RPA-CRISPR/Cas12a assay in the rapid detection of *P. ramorum*

**Figure 6:**
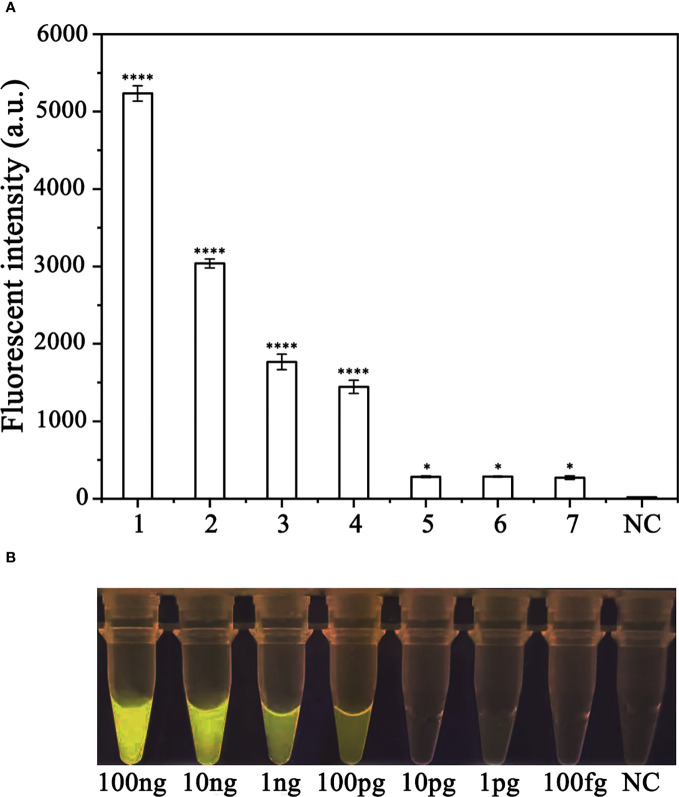
Comparison of the sensitivity of the RPA-CRISPR/Cas12a assay and conventional PCR for the detection of *Phytophthora ramorum*. **(A)** *P. ramorum* can be detected at the concentration of 100 fg using a multifunctional microplate reader (λ_ex_: 485 nm, λ_em_: 520 nm). *P <0.05 was considered statistically significant. ****P <0.0001 indicates that the difference between fluorescence and non fluorescence is very significant. **(B)** *P. ramorum*can be detected at the concentration of 100 pg using a Blue LED Transilluminator at a wavelength of 470 nm.

To assess the sensitivity of the RPA-CRISPR/Cas12a assay for detecting *P. ramorum*, different concentrations of *P. ramorum* gDNA or dd H_2_O were used as templates for the RPA reaction. The concentrations used were 100 ng, 10 ng, 1 ng, 100 pg, 10 pg, 1 pg, and 100 fg. Subsequently, 2 μl of the RPA products generated were analyzed using the CRISPR/Cas12a cleavage assay. The results showed that the samples that could be detected by a multifunctional microplate reader (λ_ex_: 485 nm, λ_em_: 520 nm) in the RPA-CRISPR/Cas12a assay were as low as 100 fg (**Figure 6A**). However, when using a Blue LED Transilluminator at a wavelength of 470 nm, the lowest detectable concentration was 100 pg of gDNA (**Figure 6B**).

### Detection of *P. ramorum* in artificially inoculated *Rhododendron × pulchrum* using RPA-CRISPR/Cas12a assay

**Figure 7:**
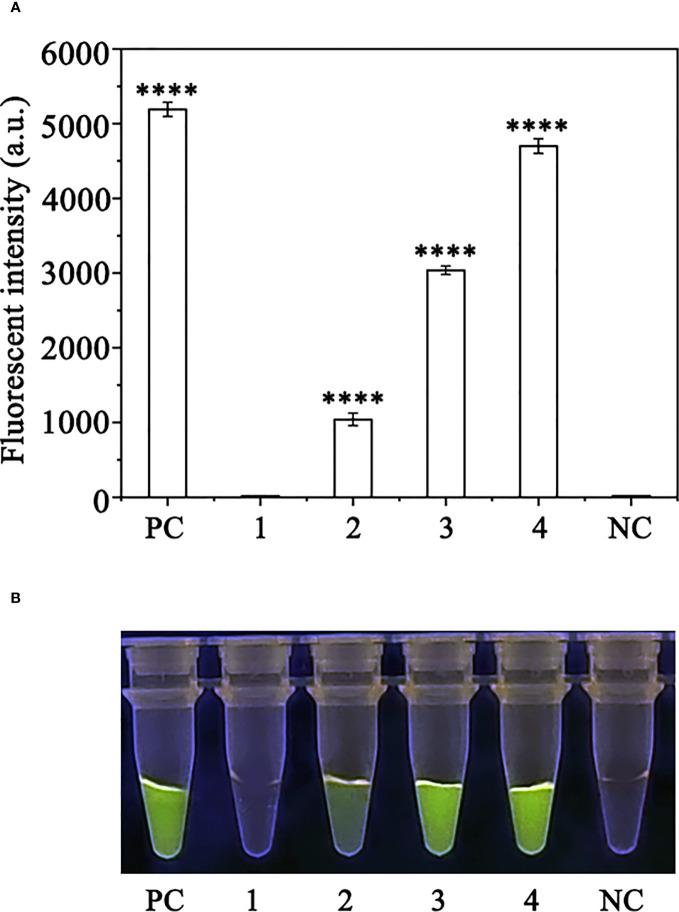
Detection of *Phytophthora ramorum* in artificially inoculated *Cedrus deodara* using the RPA-CRISPR/Cas12a assay. **(A)** Strong fluorescence signals were detected by a multifunctional microplate reader (λex: 485 nm, λem: 520 nm). PC: Positive control; 1: Control check (CK); 2-4: the first, sencond and third days of the inoculated *Rhododendron × pulchrum* leaves; NC: negative control. **(B)** Visible green fluorescence was detected under a Blue LED Transilluminator at a 470-nm wavelength. PC: Positive control; 1: Control check (CK); 2-4: the first, sencond and third days of the inoculated *Rhododendron × pulchrum* leaves; NC: negative control. ****P <0.0001 indicates that the difference between fluorescence and non fluorescence is very significant.

Crude DNA was extracted from *R. pulchrum* plants inoculated with *P. ramorum* for 1, 2, and 3 days, as well as from non-inoculated plants, using a NaOH lysis method. The extracted DNA was used as a template for the RPA-CRISPR/Cas12a assay. A positive control containing purified gDNA (100 ng) of *P. ramorum* isolates and a negative control containing ddH_2_O were also included. The RPA-CRISPR/Cas12a assay was able to detect the presence of *P. ramorum* in the crude DNA samples from the positive control and the *P. ramorum*-inoculated plants, as evidenced by green fluorescence. This was observed both under a Blue LED Transilluminator at a wavelength of 470 nm and when using a multifunctional microplate reader with excitation and emission wavelengths of 485 nm and 520 nm (**Figure 7**), respectively. No fluorescence was detected in the control check or the NTC samples.

## Discussion

*Phytophthora ramorum*, an oomycete responsible for sudden oak death in the United States and in Europe, has been found to affect over 70 species of trees and shrubs (Werres et al., 2001). *P. ramorum* causes resinous ulcers in the host trunk, necrosis in female flowers and pinecones, the spoilage of seeds, and the withering of saplings (Viljoen et al., 1994; Storer et al., 1998). Since *P. ramorum* has not been identified in China, early and accurate detection of this pathogen is crucial for effective prevention and disease management, thus avoiding unnecessary costs resulting from misdiagnosis or delayed diagnosis.

In our study, we utilized comparative whole-genome sequence analysis among *Phytophthora* species to identify a conserved region of the *Pr52094* gene that exhibited significant differences compared to other *Phytophthora* species. Based on this information, we designed RPA primers and crRNA specific for this region (**Supplementary Figure S1**). To visualize the products generated by CRISPR/Cas12a-mediated cleavage, we introduced ssDNA reporters labeled with fluorophores and quenchers (5′ 6-FAM-TTATT-BHQ-1 3′) *in vitro*, as described in previous studies (Xiong et al., 2020; Zhu et al., 2021; Li et al., 2022). We also discovered that the concentrations of crRNA and ssDNA reporters were crucial for the success of the RPA-CRISPR/Cas12a assay. To optimize the assay, we tested 11 different concentrations of crRNA and ssDNA reporters and found that the fluorescence intensity was highest at concentrations of 10 μM for both crRNA and ssDNA reporters (**Table S1**). The assay was able to distinguish *P. ramorum* from other related species, including 31 *Phytophthora* species, 5 oomycete species, 31 non-*Phytophthora* species, and 2 *Bursaphelenchus* species, by generating green fluorescence only in DNA samples obtained from *P. ramorum* (**Figure 5**). The effectiveness of the assay was also demonstrated through artificial inoculation of *P. ramorum* in plants, where DNA samples extracted using the NaOH method (Wang et al., 1993) were successfully amplified using the RPA-CRISPR/Cas12a assay. Additionally, inoculated samples, not non-inoculated ones, generated green fluorescence that could be detected under the Blue LED Transilluminator (**Figure 7**). These results indicate that the assay has high accuracy in detecting *P. ramorum* and has the potential for early diagnosis of this pathogen.

This method has several advantages compared with conventional methods. First, previous assays for detecting *P. ramorum*, such as conventional PCR (Steenkamp et al., 1999) and real-time PCR (Schweigkofler et al., 2004), were time-consuming and required expensive equipment. A more convenient and field-friendly isothermal amplification technique, LAMP, was subsequently developed but still required an amplification time of 1 hour (Stehlíková et al., 2020). But the RPA-CRISPR/Cas12a assay does not require complex equipment and has a shorter amplification time of only 25 minutes (**Figure 1**). Second, RPA-CRISPR/Cas12a reactions could be performed at 37°C, a temperature that human body temperature, USB-powered incubators or thermostatic heaters can provide. However, the detection of pathogenic bacteria, such as the PCR method, requires a higher temperature (Xiao et al., 2023). Third, this new assay also offers dual specificity using both RPA and CRISPR/Cas12a technologies. Some substances in the RPA reaction interfere with the antibodies on the test paper may causes non-specific binding and false positive signals to occur (Miao et al., 2019), which are not sufficiently diluted. Binding RPA to CRISPR-Cas12a allows us to detect the target twice, including the recognition of the RPA primer and the RPA amplification product by CRISPR-Cas12a when the RPA reaction is performed, which effectively avoids the problem of false positives during RPA amplification. Forth, roughly extracted DNA can also be used in this method without the need for high-quality and high-purity DNA which is required for conventional PCR-based diagnosis (Shin et al., 2021a), which maked it an ideal choice for establishing a rapid on-site detection technology platform. As a result, the RPA-CRISPR/Cas12a assay offers a faster and more efficient alternative to currently employed *P. ramorum* diagnostic methods.

Although these diverse properties of the CRISPR/Cas12a system provide potential for the development of versatile tools for pathogen detection (Kim et al., 2016; Li et al., 2018), there remain challenges to overcome. First, different crRNA scaffolds affect the activities of the Cas12a–crRNA complex (Li et al., 2017), which will help to elucidate the exact mechanisms of the reactions and enable improvements in the future. Secondly, the reagents required for CRISPR reactions currently need to be cryopreserved before use, which limits the application of CRISPR detection (Nguyen et al., 2021; Rybnicky et al., 2022). In the future, further exploration of the freeze-drying use of CRISPR reactions can be carried out, allowing the reagents to be stored for a long time at room temperature and truly put into large-scale application. Third, The 3′ end of crRNA is crucial for accurately interrogating DNA targets by DNA-RNA pairing. The changes and deletions of consecutive bases may affect the recognition of crRNA, thereby affecting the cleavage of Cas12a (Hu et al., 2017; Shi, 2021). Overall, the RPA-CRISPR/Cas12a assay developed in this study offers a rapid and reliable method for detecting *P. ramorum* that does not require expensive laboratory equipment, making it a promising tool for disease diagnosis and surveillance.

## Conclusions

The objective of this study was to develop a detection method for *P. ramorum* using RPA-CRISPR/Cas12a assay. Based on the experimental findings (**Figure S3**), it can be concluded that this method is highly sensitive, efficient, and convenient. It does not require complex equipment and allows for early detection and prediction of *P. ramorum*. The assay’s effectiveness makes it a viable option for improving pathogen detection and increasing sensitivity. Additionally, the RPA-CRISPR/Cas12a-based assay has the potential to be applied in areas with limited resources. This research provides a useful reference for practitioners looking to develop similar detection methods for other pathogens.

## Data availability statement

The original contributions presented in the study are included in the article/**Supplementary Materials**. Further inquiries can be directed to the corresponding authors.

## Author contributions

YG conceptualized and designed the research, analyzed the data, interpreted the results, performed the experiments and wrote the manuscript. HX participated discussed the experimental design. TD, TL, Shamoun and WC revised the manuscript and directed the project. All authors listed have made a substantial, direct, and intellectual contribution to the work and approved it for publication. All authors listed have made a substantial, direct, and intellectual contribution to the work and approved it for publication.

## Supplementary material

Supplementary Figure 1 Sequence of the *Pr52094* gene of *Phytophthora ramorum*. The nucleotides targeted by the forward (Pr52094RPA-F) and reverse (Pr52094RPA-R) primers and the sequence of the crRNA are shown below the respective arrows. Arrows indicate the direction of amplification.
