# Supplementary figures and images for: CRISPR/Cas12a-based approaches for efficient and accurate detection of Phytophthora ramorum
Source: Front Cell Infect Microbiol. 2023 Jun 27;13:1218105. doi: 10.3389/fcimb.2023.1218105 (PMC10333691; doi:10.3389/fcimb.2023.1218105)

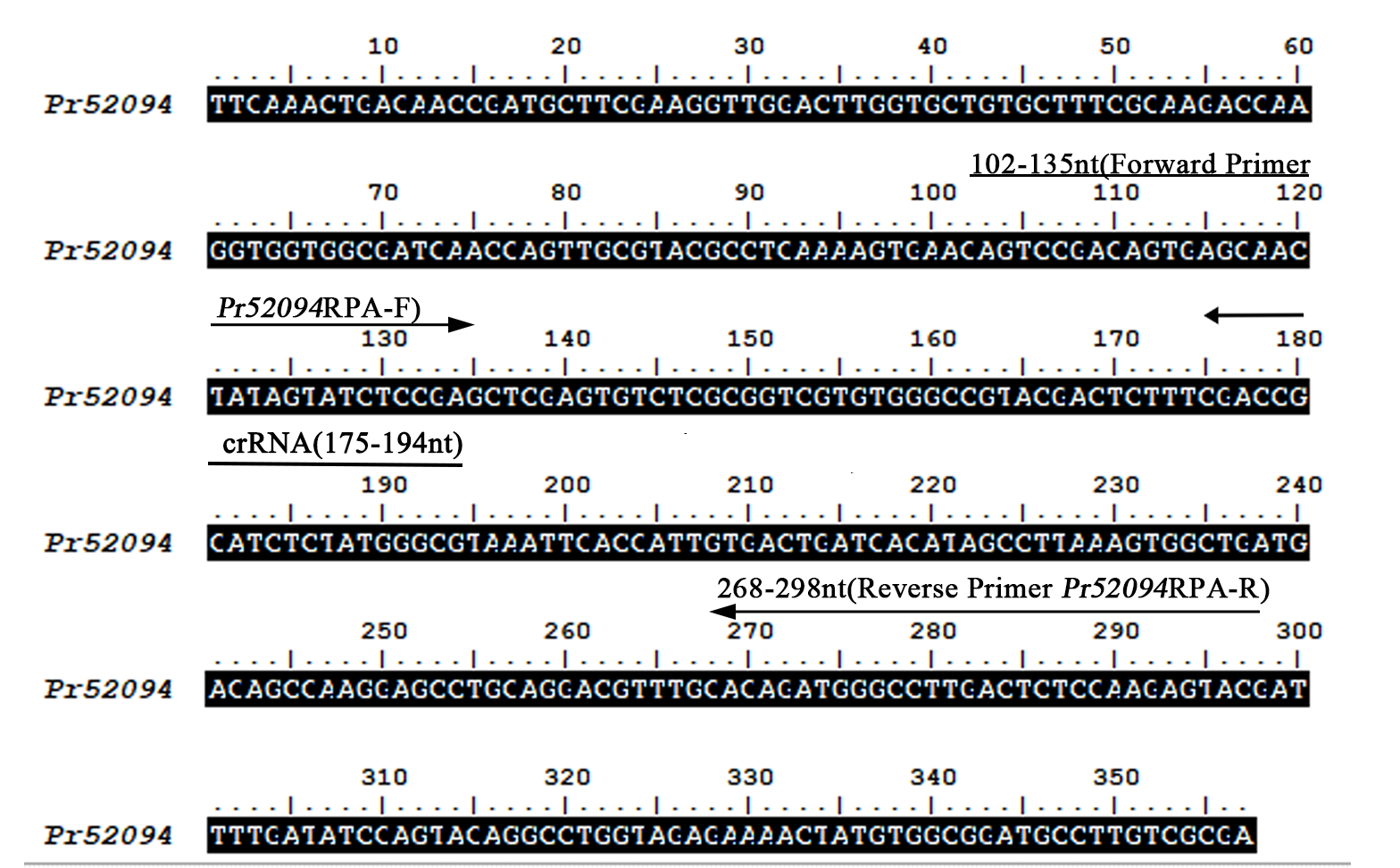

Supplement: Supplementary Figure 1 — Sequence of the Pr52094 gene of Phytophthora ramorum. The nucleotides targeted by the forward (Pr52094RPA-F) and reverse (Pr52094RPA-R) primers and the sequence of the crRNA are shown below the respective arrows. Arrows indicate the direction of amplification. [file Image_1.png]
